# New Insights into Kidney Diseases

**DOI:** 10.3390/ijms252312536

**Published:** 2024-11-22

**Authors:** Kan Katayama, Kaoru Dohi

**Affiliations:** Department of Cardiology and Nephrology, Mie University Graduate School of Medicine, Tsu 514-8507, Japan; dohik@med.mie-u.ac.jp

Chronic kidney disease (CKD) is a lifestyle-related disease affecting about 9% of the global population as of 2017 [1], and it is classified based on the degree of albuminuria/proteinuria in urine tests and the estimated glomerular filtration rate in blood tests [2]. The underlying causes include various conditions such as diabetes, hypertension, nephritis, polycystic kidney disease, transplantation, and unknown factors. In addition to existing drugs with proven nephroprotective effects, such as renin–angiotensin–aldosterone system inhibitors [3,4,5] and sodium-glucose cotransporter-2 (SGLT2) inhibitors [6,7,8], new drugs are also under development. The aim of this Special Issue is to deepen our knowledge by gathering original research articles and review articles focused on new insights into kidney diseases.

Nigro E et al. focused on the negative correlation between orexin-A levels and renal function. They analyzed orexin-A levels and the frequency of single-nucleotide polymorphisms (SNPs) in the hypocretin neuropeptide precursor (*HCRT*) and its receptors, *HCRTR1* and *HCRTR2*, in 64 patients with autosomal dominant polycystic kidney disease (ADPKD) who carry truncating mutations in their *PKD1* or *PKD2* genes. The study found that orexin-A levels in PKD patients were statistically significantly higher than those in healthy controls, and that orexin-A showed a negative correlation with blood pressure and a positive correlation with the estimated glomerular filtration rate (eGFR). However, none of the analyzed SNPs were associated with orexin-A levels. These results highlight the potential importance of orexin-A in ADPKD patients, according to the authors.

Oda K et al. focused on the involvement of the *KANK1* gene in hereditary nephrotic syndrome in humans and investigated the phenotypic changes and underlying mechanisms associated with the syndrome by generating podocyte-specific *Kank1* knockout mice. In *Kank1* knockout mice, no significant differences were observed in their urinary albumin–creatinine ratio, serum creatinine levels, or histological features compared to negative control mice. However, after adriamycin administration, the urinary albumin–creatinine ratio and sclerotic index were significantly elevated. Transmission electron microscopy revealed significantly more extensive foot process effacement in knockout mice compared to negative control mice. Furthermore, *KANK1*-deficient human podocytes showed significantly increased detachment and apoptosis upon exposure to adriamycin compared to wild-type podocytes. Based on these findings, the authors suggested that *KANK1* may play a protective role in mitigating podocyte damage under pathological conditions.

Bărar AA et al. used glomerular proteomic analysis to elucidate the mechanisms of podocyte dysfunction in minimal change disease (MCD). Glomeruli from formalin-fixed paraffin-embedded (FFPE) renal biopsy samples were sectioned by laser-capture microdissection (LCM) and shotgun proteomics was performed to identify 48 proteins that were differentially expressed in the negative control and MCD groups. These included proteins involved in focal adhesion (NID1 and ITGA3) and slit diaphragm signaling (ANXA2, TJP1 and MYO1C), as well as components of podocyte actin and microtubule cytoskeleton (ACTR3 and NES). These results suggest that mass spectrometry-based shotgun proteomic analyses using LCM glomeruli may provide valuable insights into the etiology of podocyte diseases such as MCD.

Pavlakou P et al. focused on the changes in keratin expression patterns in epithelial cells under stress stimuli and investigated the expression of keratin in the glomeruli and tubules of patients with podocytopathy, anti-neutrophil cytoplasmic antibody (ANCA)-associated vasculitis, and IgA nephropathy, examining how this expression correlates with clinical outcomes. In biopsy sections, the expression of keratins 7, 8, 18, and 19 was analyzed separately in the glomerular and tubulointerstitial regions to assess their correlation with long-term renal function outcomes. All four keratins examined showed significantly increased glomerular expression in ANCA vasculitis patients compared to controls and MCD patients. The tubular expression of keratins 7, 8, and 19 was associated with kidney outcomes across all groups. Based on these findings, the authors suggested that keratins could be promising biomarkers for predicting renal function in patients with glomerular diseases.

Gajewska A et al. reviewed the evidence on the renoprotective, anti-inflammatory, and anti-fibrotic effects of SGLT2 inhibitors. They also noted that meta-analyses have shown the effectiveness of SGLT2 inhibitors in kidney diseases, particularly those caused by diabetes.

Righini M et al. reviewed extrarenal involvement in ADPKD. ADPKD is the most common hereditary kidney disease and is a systemic condition that involves not only the kidneys but also cystic lesions in the liver, pancreas, and brain, as well as non-cystic lesions in the blood vessels, gastrointestinal tract, bones, and heart valves. The review summarizes how liver lesions and vascular abnormalities significantly impact the quality of life and prognosis of ADPKD patients and highlights recent findings indicating that bone disease may also have a serious effect.

Ponticelli C et al. reviewed the role of autophagy in ADPKD. Recently, autophagy has emerged as a potential contributor to the pathogenesis of ADPKD, and this review focuses on the complex involvement of autophagy in ADPKD. Promising results have been observed with autophagy-inducing agents in preclinical trials, and the authors conclude that clinical trials are needed to thoroughly evaluate the long-term safety and efficacy of combining autophagy-inducing agents with metabolic and/or aquaferetic drugs.

This Special Issue features a wide range of papers, including basic research on nephrotic syndrome, studies using glomerular proteomics, clinical research on samples from ADPKD patients, and research on keratin in various kidney diseases. Its review articles provide an in-depth look at the latest insights on SGLT2 inhibitors, as well as a deeper understanding of extrarenal manifestations and autophagy in ADPKD. We are grateful to all the authors who contributed to this Special Issue and hope that it will promote further advances in our understanding of the mechanisms of kidney diseases and in the development of new therapeutic agents.
